# A multi-level analysis of the relationship between environmental factors and questing *Ixodes ricinus* dynamics in Belgium

**DOI:** 10.1186/1756-3305-5-149

**Published:** 2012-07-25

**Authors:** Sen Li, Paul Heyman, Christel Cochez, Leopold Simons, Sophie O Vanwambeke

**Affiliations:** 1 Georges Lemaître Centre for Earth and Climate Research, Earth and Life Institute, Université catholique de Louvain, B-1348, Louvain-la-Neuve, Belgium; 2 Research Laboratory for Vector Borne Diseases, Queen Astrid Military Hospital, B-1120, Brussels, Belgium

**Keywords:** *Ixodes ricinus*, Spatio-temporal dynamics, Multi-level analysis, Environment, Belgium

## Abstract

**Background:**

Ticks are the most important pathogen vectors in Europe. They are known to be influenced by environmental factors, but these links are usually studied at specific temporal or spatial scales. Focusing on *Ixodes ricinus* in Belgium, we attempt to bridge the gap between current “single-sided” studies that focus on temporal or spatial variation only. Here, spatial and temporal patterns of ticks are modelled together.

**Methods:**

A multi-level analysis of the *Ixodes ricinus* patterns in Belgium was performed. Joint effects of weather, habitat quality and hunting on field sampled tick abundance were examined at two levels, namely, sampling level, which is associated with temporal dynamics, and site level, which is related to spatial dynamics. Independent variables were collected from standard weather station records, game management data and remote sensing-based land cover data.

**Results:**

At sampling level, only a marginally significant effect of daily relative humidity and temperature on the abundance of questing nymphs was identified. Average wind speed of seven days prior to the sampling day was found important to both questing nymphs and adults. At site level, a group of landscape-level forest fragmentation indices were highlighted for both questing nymph and adult abundance, including the nearest-neighbour distance, the shape and the aggregation level of forest patches. No cross-level effects or spatial autocorrelation were found.

**Conclusions:**

Nymphal and adult ticks responded differently to environmental variables at different spatial and temporal scales. Our results can advise spatio-temporal extents of environment data collection for continuing empirical investigations and potential parameters for biological tick models.

## Background

*Ixodes ricinus* is a hard tick species (Acari: Ixodidae) widely distributed in Europe. It is capable of transmitting a number of pathogens to humans and livestock. For example, Lyme borreliosis, caused by the bacterium *Borrelia burgdorferi,* is the most frequent tick-borne disease of humans in temperate zones [1]. Louping ill, an acute viral disease primarily of sheep, can result in a mortality of up to 60% for infected sheep. Understanding the spatio-temporal dynamics of *I. ricinus* can be of public health and economic importance.

The current literature on tick patterns documents two types of associations: (i) between the extrinsic climatic factors and temporal dynamics and (ii) between the physical/biotic environmental factors and spatial dynamics. Studies on the temporal dynamics of ticks generally investigate tick phenology, which refers to the seasonal cycle of development and activity of the life stages of tick and the impacts from seasonal and inter-annual climate variations. Tick survival and development rates can be influenced by relative humidity and temperature [2,3] and the host-seeking activity can be affected by day length and vapour pressure deficit [4]. Kurtenbach et al. [5] proposed that the phenology of ticks shapes the temporal patterns of tick-borne diseases. Studies on the spatial dynamics of ticks often focus on the suitability of habitat. Preferential habitats of ticks and many of their hosts occupy a large fraction of rural landscapes. Habitat types, regarding the landscape composition, landscape configuration, soil conditions, bedrock geology, topography, etc., have been largely and empirically tested for their associations to the dynamics of ticks. Forests are the most suitable habitats for ticks in most studies in North America (*I. scapularis*) and Europe (*I. ricinus*) [6]. Their configurations have been identified as key determinants of the spatial dynamics of ticks [7,8]. The abundance of questing (*I. scapularis*) ticks is positively associated with sandy or sandy loam soils and sedimentary bedrock [9]. In the Czech Republic [10], Switzerland [11] and Spain [12], topographic variables also potentially influence the local tick abundance. Landscape can shape the patterns of host dynamics. Key hosts such as bank voles and deer inhabit agrarian and forested landscape [5]. Moreover, ecotones between forest and cultivated fields, pastures and other grassland areas can be of high value to ticks and some host species [13]. The relationship between tick abundance and landscape structure, however, has been sparsely investigated.

However, current investigations suffer from several limitations. Firstly, researchers have rarely investigated tick patterns both temporally and spatially. One study examined the influence of weather and biotic (host) factors in northern Italy [14], but did not take physical environmental factors into account. Secondly, there is limited understanding of the influence of forest fragmentation on the spatial dynamics of ticks. A recent regional level study demonstrated a positive effect of forest edge on questing tick abundance in northern Belgium [15]. Forest patch size was also found negatively associated to (*I. scapularis*) tick abundance in southeastern New York [8]. In northern Spain, high tick abundance was found in ‘stepping-stone’ patches that cause significant changes in connectivity when removed from the landscape [7]. Other than these patch-level effects, a number of landscape-level characteristics describing the spatial configuration of patches (e.g. neighbouring distance, aggregation level, fractal dimension) can be also important as they may shape the local host exchange patterns. Thirdly, the influence of wildlife management needs to be further examined. Effects of deer exclusion by fencing have been empirically studied in Scotland [16] and Italy [17]. Hunting, however, has received less attention thus far. Hunting can directly influence the local populations of large vertebrates (e.g. deer), which are key reproduction hosts for adult female ticks. It can be important to explore whether hunting can be used as a strategy to reduce the local tick abundance, provided the spatial dataset of wildlife are sometimes too difficult to get.

To address these limitations, a multi-level analysis was carried out to study the spatio-temporal dynamics of *I. ricinus* abundance in Belgium. A multi-level approach is considered suitable for the present study because of the clustered nature of our data, i.e. repeated tick samplings in most individual sites. Despite a growing interest in this approach in the field of vector-borne diseases [18], it has, to our knowledge, never been used in previous tick-related studies. In the present study, joint effects of weather, habitat quality and wildlife management factors were examined at two levels: (i) sampling level, which is associated with temporal tick dynamics, and (ii) site level, which is related to spatial tick dynamics. The main objective was to determine in each level the key factors and to examine possible cross-level effects to the spatio-temporal dynamics of questing ticks in Belgium.

## Methods

### Study sites

**Figure 1:**
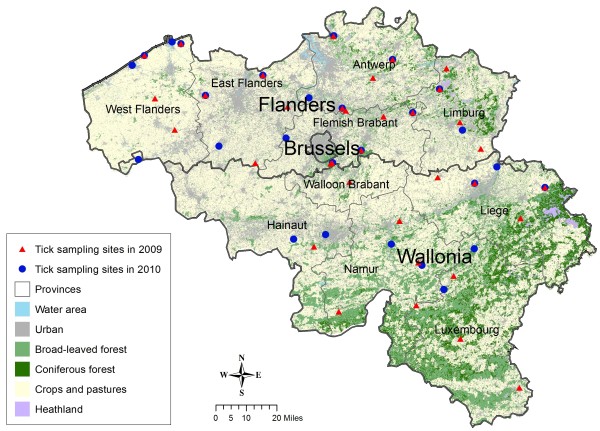
Map of Belgium and location of sampling sites in 2009 and 2010.

From 2009 to 2010, 125 samplings were carried out in 51 sites throughout Belgium (Figure 1): 36 sites were visited in 2009 and 27 sites were visited in 2010, among which 12 sites were visited in both years. Sites were initially selected based on a 25 km * 25 km regular grid across Belgium, from which unsuitable habitats (such as built-up areas) were excluded. In the field, some sites were moved, in order to be placed in a suitable forest stand. All sampling sites were in broad-leaved or mixed forest stands. Most of these forest stands are adjacent to cropland and pasture only, while a few of them were also adjacent to urban and water areas.

### *Ixodes ricinus* sampling scheme

Standard blanket-dragging was adopted to estimate the abundance of the questing ticks from March to November in 2009 and from March to September in 2010. These timings were selected because the ‘tick season’ generally lasts from March to October in Belgium [19]. During each sampling, a 1 m² piece of white flannel was pulled over the short grass or litter along a 500 m transect. High ground vegetation such as purple moor grass and bracken fern was avoided. The blanket was examined every 20 m and nymphs and adults were preserved in alcohol for future laboratory identification. The identification was done on the basis of morphologic features with a stereo-microscope by using standard taxonomic keys [20]. All samplings were performed between 9:30 and 13:00 and rainy days were excluded. At the beginning of each sampling, coordinates were recorded by military GPS (spatial resolution 1 m).

### Environmental variables

Environmental variables were collected at two levels: (i) at the level of sampling event (“sampling level”), focusing on the influence of seasonal weather variations on the temporal patterns of ticks; (ii) at site level, considering the role of landscape (composition and configuration) and soil conditions on the quality of tick habitat, as well as hunting activity that affects the population of host animals.

Weather data was acquired from the Royal Institute of Meteorology for all working stations covering the period of sampling. Measures were available from 50 meteorological stations for daily maximum and minimum temperatures and precipitation and from 25 stations for daily wind speed and relative humidity. The average distance between sampling sites and the closest station for temperature and precipitation is 17.3 km and that for wind speed and relative humidity is 12.3 km. For each sampling session, we selected the nearest meteorological stations for daily maximum and minimum temperatures, precipitation, wind speed and relative humidity. In addition, average daily vapour pressure deficits (VPD) were estimated from observations of temperature and relative humidity, following [21]: *VDP* = *es*(*Ta*) × (1–*RH*), where *es*(*Ta*) is the saturation vapour pressure function of mean temperature *Ta* and *RH* is the relative humidity. Mean values of these variables in 3 and 7 days prior to the sampling day were also calculated.

A land cover classification was generated based on Landsat satellite images (combined from two captures on 2000/09/11 and 2001/05/23; resolution 29.18 m). Landscape composition was measured in 500 m-, 1000 m- and 1500 m- buffers around each sampling site. The radius of 500 m was chosen because it was the total length for one field sampling. Radii of 1000 m and 1500 m were selected to examine the landscape effects at larger extents. The proportions of potential habitat types (i.e. broad-leaved forest, coniferous forest, heathland and agricultural areas of crops and pastures) were calculated. The configuration of forests was examined within the same buffers using several indices [22]: the number of forest patches, edge density (the sum of the lengths of all edge segments involving the forest type divided by the total landscape area), aggregation index (aggregation levels of forest patterns), Euclidean mean nearest-neighbour distance (mean distance to the nearest neighbouring forest patches), area-weighted mean patch fractal dimension (overall complexity and fragmentation of forest patches) and area-weighted mean shape index (overall shape complexity of forest patches).

Two digital soil maps, for Wallonia (by “Unité Sol-Ecologie-Territoire, FUSAGx”) and for Flanders (by “Vlaamse Landmaatschappij”), were acquired. Information on soil texture and drainage was extracted from both maps. For each sampling site, soil textures were coded as percentages of sand, silt, clay and gravel. Drainage conditions were indexed from 0 to 8 from excessive to poor drainage across Belgium, while values of only 0 to 6 were observed for the sampling sites.

Data on roe deer shot in 2009 were collected. In Wallonia, data was provided for each forest-management administrative unit (i.e. “cantonnement”) by “Direction Générale des Ressources Naturelles et de l’Environnement”. In Flanders, data was provided by “Instituut voor Natuur– en Bosonderzoek” and recorded in each administrative unit (i.e. “wildbeheereenheden”, a unit that is much smaller than “cantonnement”). For each administrative unit (“cantonnement” for Wallonia or “wildbeheereenheden” for Flanders), numbers of roe deer shot were divided by the forested area to get the number of shot roe deer per km² of forest. The data may be biased as officials from the two regions focused on different spatial scales and may put different weights on information sources when making estimations. To weaken the effects of such uncertainty, we classified the number of roe deer shot per km² of forest into five categories using an interval of 50.

### Statistical analysis

The abundances of nymphs and adults were analysed separately using multi-level approaches. All statistical analyses were conducted with R (version 2.14.1) [23].

In the first step, a test for overdispersion (i.e. likelihood ratio of a fitted Poisson model against a fitted negative binomial model) indicated that the data was overdispersed and that using a negative binomial distribution was necessary. A non-parametric Kruskal-Wallis one-way analysis of variance was used to test the necessity to use random intercept models. The between-site variance of intercepts was significantly different from zero (nymphs: chi-squared = 89.3, df = 50, P < 0.001; adults: chi-squared = 83.5, df = 50, P < 0.005). Thus, models that allow for random intercepts are better suited than those with fixed intercepts.

In the second step, single level regressions (i.e. bivariate negative binomial regressions) were performed to select significant variables (P < 0.1) for each level. At sampling level, the dependent variable was the tick sampling result per site and the weather data were examined as independent variables. At site level, the dependent variable was defined as the mean value of sampled ticks in each site. Landscape composition and configuration, soil and hunting data were analysed as independent variables.

In the third step, a set of multi-level negative binomial models with random intercepts were developed for model selection. Instead of evaluating a single model, a model selection approach offers a way to draw inferences from competing models by evaluating their relative support in the data [24]. An Automatic Differentiation (AD) model builder approach in R, package glmmADMB [25], was used to build the multi-level models. We generated nine groups of models by adding up to two variables for each level. For each group, models were built in an automated fashion by looping all combinations of variables selected in the second step. Models were rejected if not all predictors were significant at <0.1 or if significant colinearity was observed (Pearson correlation coefficients between independent variables >0.7 and P < 0.1) among variables. We sorted the remaining models by their improvements on the model fit in comparison to intercept-only models as assessed by the change in Akaike’s Information Criterion (AIC) (ΔAIC = AIC of intercept-only model–AIC of model with variables). Variables were ranked according to their relative importance (by summing the Akaike weights across the models that include the variable [26]) in the above-average models (i.e. models for which ΔAICs > averaged ΔAIC value of the remaining models). The residuals of the top five models with the greatest model fitness improvement (the largest ΔAICs) were checked for spatial autocorrelation in model residuals, the significance of random slope and cross-level effects.

## Results

### Spatio-temporal variation of questing nymphs and adults

A total of 22 400 *I. ricinus* ticks were collected, including 20 725 nymphs and 1 675 adults (828 males and 847 females). More samplings were conducted in Flanders (93 times) than in Wallonia (32 times). Detailed seasonal patterns of sampling for each province are presented in Table 1. In general, *I. ricinus* ticks were found in all ten provinces in Belgium. Tick abundance per sampling was calculated for all ten provinces: in Flanders the sampled tick abundance was higher in East Flanders, Flemish Brabant and Limburg; while in Wallonia, that was higher in Namur, Hainaut and Luxembourg.

**Table 1:** ***Ixodes ricinus*sampled between 2009 and 2010 from ten provinces in Belgium (S = number of sampling; N.N = number of nymphs; N.A = number of adults; “-” = not sampled)**

**Province**		**Year (20-)**	**Spring (Mar–May)**			**Summer (Jun–Aug)**			**Autumn (Sep–Nov)**			**Nymphs per 100 m²**	**Adults per 100 m²**
			**S**	**N.N**	**N.A**	**S**	**N.N**	**N.A**	**S**	**N.N**	**N.A**		
Flanders	Antwerp	09	4	627	103	1	245	15	3	194	14	26.7	3.3
		10	3	513	22	4	578	82	2	217	16	29.1	2.7
	East Flanders	09	4	219	84	2	427	50	–	–	–	21.5	4.5
		10	5	1683	47	2	541	32	–	–	–	63.5	2.3
	Flemish Brabant	09	10	2769	278	6	2393	109	11	1458	204	49.0	4.4
		10	8	2420	161	6	1802	44	2	529	30	59.4	2.9
	Limburg	09	2	153	16	1	298	45	2	334	37	31.4	3.9
		10	1	455	14	3	818	33	1	142	7	56.6	2.2
	West Flanders	09	2	0	1	2	23	8	–	–	–	1.2	0.5
		10	3	2	1	3	3	0	–	–	–	0.2	0.0
Wallonia	Hainaut	09	–	–	–	1	125	3	–	–	–	25.0	0.6
		10	3	65	11	2	5	1	–	–	–	2.8	0.5
	Liege	09	1	10	0	3	144	8	–	–	–	7.7	0.4
		10	3	140	9	1	26	1	–	–	–	8.3	0.5
	Luxembourg	09	1	46	10	3	196	21	–	–	–	12.1	1.6
		10	1	86	3	–	–	–	–	–	–	17.2	0.6
	Namur	09	3	107	33	1	42	9	1	33	4	7.3	1.8
		10	4	397	30	2	295	16	1	80	12	22.1	1.7
	Walloon Brabant	09	1	85	51	–	–	–	–	–	–	17.0	10.2
		10	–	–	–	–	–	–	–	–	–	–	–
All (ten) provinces		09	28	4016	576	20	3893	268	17	2019	259	30.5	3.4
		10	31	5761	298	23	4068	209	6	968	65	36.0	1.9

**Figure 2:**
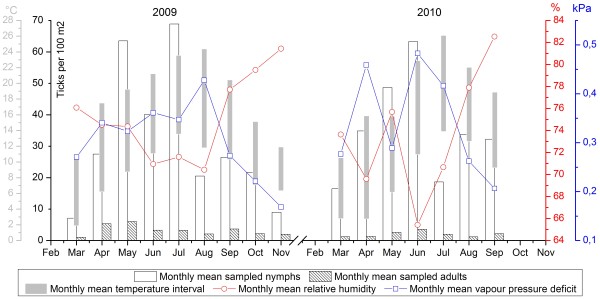
**Temporal variation of ticks in 2009 and 2010.** Red and blue lines indicate changes of monthly mean relative humidity and vapour pressure deficit. Grey bars indicate the interval between maximum and minimum temperatures. Mean values of ticks were calculated per 100 m².

The temporal variation of ticks in 2009 and 2010 is presented in Figure 2. In both years, the abundance of nymphs started to increase in spring, then fluctuated and reached a first peak in summer (July for 2009 and June for 2010)–one month before the hottest month. The second peak came in late summer and early autumn–one month after the hottest month. The association between the sampling results and meteorological conditions appears to be varying throughout the two sampling years. In both springs, questing tick abundance increased while temperature increased. The decline of the nymph abundance in the summer of 2009 can be associated to a peak in vapour pressure deficit or to a drop in maximal relative humidity. In the summer of 2010, nymph abundance peaked while vapour pressure deficit peaked and relative humidity dropped. Finally, in both autumns, questing tick abundance decreased while temperature and vapour pressure deficit decreased but relative humidity increased.

### Single level regressions

Results for regressions at sampling level and for regressions at site level are presented in Tables 2 and 3 together with descriptive statistics of the collected variables. At sampling level, abundance of nymphs was found significantly associated with maximum temperature, relative humidity and vapour pressure deficit on the sampling day. Vapour pressure deficit 3 and 7 days prior to sampling was also significant but at a lower level. The influence of wind speed was only significant at the greatest temporal scale considered i.e. 7 previous days. The abundance of adults was significantly correlated only to wind speed averaged for 7 previous days. At site level (Table 3), both mean abundances of nymphs and adults were found significantly associated with the proportions of forest area and a set of landscape configuration measures including forest edge density, shape index, nearest neighbour distance and aggregation index of forest patches. As the buffer radius increased, the significance of all landscape composition and configuration characteristics increased. Site mean abundance of nymphs was also found negatively related to the percentage of clay in the soil and the abundance categories of roe deer shot.

**Table 2:** Sampling level bivariate regressions

**Variables**		**Descriptive Statistics**		**Coefficient (Std. Error) in Regressions**	
		**Mean**	**Range**	**Nymph abundance**	**Adult abundance**
Daily minimum temperature (°C)	Sampling day	7.97	−2.40–16.80	0.03 (0.03)	0.01 (0.02)
	3 previous days mean	8.17	0.03–18.07	0.05 (0.03)	0.03 (0.03)
	7 previous days mean	8.21	−1.20–18.56	0.05 (0.03)	0.03 (0.03)
Daily maximum temperature (°C)	Sampling day	19.55	8.40–33.00	0.05 (0.03)*	0.02 (0.02)
	3 previous days mean	18.73	7.40–29.80	0.04 (0.03)	0.01 (0.02)
	7 previous days mean	18.44	5.16–29.64	0.03 (0.03)	0.01 (0.02)
Daily precipitation (mm)	Sampling day	1.28	0–24.30	−0.02 (0.04)	−0.02 (0.03)
	3 previous days mean	1.44	0–21.47	−0.02 (0.05)	−0.04 (0.05)
	7 previous days mean	1.93	0–11.79	0.04 (0.06)	−0.01 (0.05)
Daily wind speed (m/s)	Sampling day	3.13	0.90–8.00	−0.14 (0.10)	−0.14 (0.09)
	3 previous days mean	3.19	0.90–7.60	−0.12 (0.13)	−0.10 (0.11)
	7 previous days mean	3.27	1.17–8.99	−0.49 (0.14)***	−0.26 (0.12)*
Daily relatively humidity (%)	Sampling day	71.75	52.00–93.00	−0.04 (0.01)*	−0.01 (0.01)
	3 previous days mean	74.36	47.67–94.67	−0.03 (0.02)	−0.01 (0.01)
	7 previous days mean	74.19	51.00–91.86	−0.03 (0.02)	−0.001 (0.02)
Daily vapour pressure deficit (kPa)	Sampling day	0.47	0.11–1.25	1.37 (0.55)*	0.41 (0.48)
	3 previous days mean	0.42	0.07–1.35	**1.09 (0.62).**	0.18 (0.53)
	7 previous days mean	0.42	0.12–1.17	**1.19 (0.67).**	0.06 (0.58)

Significant variables are highlighted in bold.

***P < 0.001; **P < 0.01; *P < 0.05; .P < 0.1.

**Table 3:** Site level bivariate regressions

**Variables**		**Descriptive Statistics**		**Coefficient (Std. Error) in Regressions**	
		**Mean**	**Range**	**Site mean nymph abundance**	**Site mean adult abundance**
*Landscape composition variables*					
Proportion of broad-leaved forest (%)	r^a^ = 500 m	24.98	0–94.95	2.84 (0.99)**	1.67 (0.82)*
	r = 1000 m	21.66	0.88–80.30	4.14 (1.13)***	2.75 (0.93)**
	r = 1500 m	21.27	1.06–85.32	4.61 (1.14)***	3.62 (0.91)***
Proportion of coniferous forest (%)	r = 500 m	2.13	0–29.18	5.89 (5.01)	3.67 (4.03)
	r = 1000 m	3.92	0–86.68	1.13 (1.97)	0.63 (1.60)
	r = 1500 m	2.50	0–25.16	14.61 (5.52)**	10.25 (4.42)*
Proportion of crops and pastures (%)	r = 500 m	35.25	0–89.34	−0.37 (1.00)	−0.10 (0.82)
	r = 1000 m	35.72	0.11–77.07	−0.31 (1.25)	−0.03 (1.02)
	r = 1500 m	40.10	6.70–83.70	−0.61 (1.36)	−0.90 (1.10)
Proportion of heathland (%)	r = 500 m	0.11	0–1.11	1.78 (102.86)	−16.94 (84.09)
	r = 1000 m	0.14	0–1.19	−31.32 (109.82)	−23.92 (89.91)
	r = 1500 m	0.34	0–11.28	−34.64 (16.62)*	−18.81 (14.88)
*Landscape (forest) configuration variables*					
The number of forest patches	r = 500 m	11.16	0–27.00	−0.03 (0.04)	0.002 (0.03)
	r = 1000 m	51.47	6.00–104.00	0.001 (0.01)	0.005 (0.008)
	r = 1500 m	124.90	12.00–240.00	0.001 (0.004)	0.002 (0.003)
Forest edge density (m/ha)	r = 500 m	21.56	0–76.97	0.03 (0.01)*	**0.02 (0.01).**
	r = 1000 m	9.83	0–48.32	0.06 (0.02)***	0.06 (0.01)***
	r = 1500 m	14.12	0–47.72	0.07 (0.02)***	0.06 (0.01)***
Area-weighted mean forest shape index	r = 500 m	2.06	0–3.5	0.33 (0.37)	0.42 (0.30)
	r = 1000 m	2.67	1.02–4.25	0.66 (0.26)*	0.79 (0.20)***
	r = 1500 m	3.01	1.05–5.13	0.72 (0.19)***	0.80 (0.14)***
Area-weighted mean forest patch fractal dimension	r = 500 m	1.11	0–1.22	0.01 (1.50)	0.49 (1.24)
	r = 1000 m	1.15	1.02–1.23	10.47 (4.32)*	13.13 (3.45)***
	r = 1500 m	1.16	1.03–1.24	11.94 (3.77)**	14.71 (2.99)***
Euclidean mean nearest-neighbour distance between forest patches (m)	r = 500 m	102.99	0–589.62	−0.02 (0.01)**	**−0.005 (0.003).**
	r = 1000 m	89.03	63.88–339.93	−0.04 (0.01)**	−0.08 (0.04)*
	r = 1500 m	88.64	63.81–195.46	−0.04 (0.01)***	−0.03 (0.01)***
Aggregation index of forest patches	r = 500 m	63.13	0–94.25	0.02 (0.01)	0.01 (0.01)
	r = 1000 m	59.42	23.94–89.51	0.04 (0.01)**	0.04 (0.01)***
	r = 1500 m	57.67	24.22–90.23	0.04 (0.01)***	0.05 (0.01)***
*Soil variables*					
Drainage degree		2.98	0–6	−0.17 (0.17)	0.06 (0.14)
Proportion of gravel (%)		3.92	0–20.00	−1.51 (3.08)	−1.40 (2.52)
Proportion of sand (%)		37.35	0–90.00	1.01 (0.72)	0.74 (0.59)
Proportion of slit (%)		41.92	0–85.00	−0.25 (0.86)	−0.14 (0.70)
Proportion of clay (%)		10.92	0–50.00	−5.12 (2.45)*	−3.18 (2.05)
*Wildlife variables*					
Categories of shot roe deer number per 1 km² forest		1.98	1.00–5.00	**−0.31 (0.18).**	−0.20 (0.15)
Number of shot roe deer per 1 km² forest		12.09	0–47.29	−0.02 (0.01)	−0.01(0.01)

Significant variables are highlighted in bold.

^a^ r : Buffer zone radius.

***P < 0.001; **P < 0.01; *P < 0.05; .P < 0.1.

### Multi-level regressions

Variables with P < 0.1 in single-level bivariate regressions were selected for multi-level regressions. A total number of 4 201 models for nymphs and 273 models for adults were generated by adding up to two variables for each level. Models containing significant collinearity and any non-significant covariates were removed, leaving 258 models for nymph abundance and 60 models for adult abundance. Based on their improvement on model fitness (i.e. ΔAIC), there were 136 and 29 above-average models for nymphs and adults respectively.

The relative importance of variables is presented in Table 4. The highest summed value of Akaike weights in above-average models of nymph abundance were reached by, at sampling level, daily relative humidity, mean value of daily wind speed in 7 previous days and daily maximum temperature and by, at site level, euclidean mean nearest-neighbour distance, area-weighted mean forest shape index and area-weighted mean patch fractal dimension. The most important predictors of adult abundance were the mean value of daily wind speed in 7 previous days at sampling level and area-weighted mean patch fractal dimension, forest edge density, euclidean mean nearest-neighbour distance and proportion of broad-leaved forest at site level.

**Table 4:** Relative importance of variables (Sum of Akaike weights >0.1) in above-average models

**Level**	**Variable**	**Sum of Akaike weights**
*Key determinants to nymph abundance:*		
Sampling level	Daily relative humidity (sampling day)	0.56
	Daily wind speed (7 previous days mean)	0.31
	Daily maximum temperature (sampling day)	0.16
Site level	Euclidean mean nearest-neighbour distance (r^a^ = 500 m)	0.99
	Area-weighted mean forest shape index (r = 1500 m)	0.70
	Area-weighted mean patch fractal dimension (r = 1500 m)	0.24
*Key determinants to adult abundance:*		
Sampling level	Daily wind speed (7 previous days mean)	0.77
Site level	Area-weighted mean patch fractal dimension (r = 1500 m)	0.84
	Forest edge density (r = 1500 m)	0.34
	Euclidean mean nearest-neighbour distance (r = 1500 m)	0.22
	Proportion of broad-leaved forest (r = 1500 m)	0.13
	Forest edge density (r = 1000 m)	0.11

^a^ r : Buffer zone radius.

For all the top five nymph and adult models, neither spatial autocorrelation in residuals nor significant random slopes and cross-level effects were found. The between-site variances were greatly reduced in models with parameters than in the intercept-only model (model 0, Tables 5 and 6). By including the site-level variables, site-level variances in the best models (model 1, Tables 5 and 6) for nymph abundance and for adult abundance have respectively dropped by 75.9% and 84.5%. These findings indicate that the variability of nymph and adult tick abundance among sites can be adequately explained by the site-level variables included in the top models.

**Table 5:** Null model and the top five models for nymph abundance

	**Model 0**	**Model 1**	**Model 2**	**Model 3**	**Model 4**	**Model 5**
**Fixed effect**	Coefficient (Std. Error)					
Intercept	3.29 (0.39)***	3.77 (1.14)***	3.94 (1.38)**	1.87 (1.05).	−13.29 (5.35)*	−16.28 (5.60)**
Level–1 : Sampling						
Daily wind speed (7 previous days mean)		−0.33 (0.01)*			−0.30 (0.15)*	
Daily relative humidity (sampling day)			−0.02 (0.01).		−0.02 (0.01).	−0.02 (0.01).
Daily maximum temperature (sampling day)				0.03 (0.02).		
Level–2 : Site						
Euclidean mean nearest-neighbour distance (r^a^ = 500 m)		−0.02 (0.01)***	−0.02 (0.00)***	−0.02 (0.01)*	−0.03 (0.01)***	−0.03 (0.01) **
Area-weighted mean forest shape index (r = 1500 m)		1.01 (0.21)***	1.10 (0.23)***	1.06 (0.22)*		
Area-weighted mean patch fractal dimension (r = 1500 m)					18.63 (4.32)***	20.50 (4.65).
**Random effect**	Variance (Std. Error)					
Intercept	5.46 (0.21)	1.32 (0.10)	2.00 (0.13)	1.73 (0.12)	1.62 (0.11)	2.17 (0.13)
**AIC**	1433.25	1391.81	1393.49	1393.92	1394.68	1396.54
**ΔAIC**	0	41.44	39.76	39.33	38.56	36.71

^a^ r : Buffer zone radius.

***P < 0.001; **P < 0.01; *P < 0.05; .P < 0.1.

**Table 6:** Null model and the top five models for adult abundance

	**Model 0**	**Model 1**	**Model 2**	**Model 3**	**Model 4**	**Model 5**
**Fixed effect**	Coefficient (Std. Error)					
Intercept	1.65 (0.27)***	−12.95 (3.53)***	−12.95 (2.69)***	−13.61 (3.70)***	−9.62 (5.50).	−17.08 (3.67)***
Level–1 : Sampling						
Daily wind speed (7 previous days mean)		−0.23 (0.14).	−0.31 (0.12)**	−0.27 (0.15).	−0.23 (0.13).	
Level–2 : Site						
Proportion of broad-leaved forest (r^a^ = 1500 m)			1.18 (0.44)**			−0.24 (0.13).
Forest edge density (r = 1000 m)				0.02 (0.01).		
Forest edge density (r = 1500 m)		0.03 (0.02)*				
Area-weighted mean patch fractal dimension (r = 1500 m)		12.94 (3.04)*	13.46 (2.32)***	13.71 (3.10)***	11.76 (4.07).	16.85 (3.03) ***
Euclidean mean nearest-neighbour distance (r = 1500 m)					−0.02 (0.01)***	
**Random effect**	Variance (Std. Error)					
Intercept	1.71 (0.12)	0.26 (0.05)	1.07e−07 (2.93e-5)	0.27 (0.05)	0.33 (0.05)	0.47 (0.06)
**AIC**	881.85	853.93	855.50	855.78	856.05	856.76
**ΔAIC**	0	27.92	26.35	26.07	25.80	25.09

^a^ r : Buffer zone radius.

***P < 0.001; **P < 0.01; *P < 0.05; .P < 0.1.

## Discussion

Human risk of contracting tick-borne diseases is directly linked to the timing of the questing activity and the distribution of ticks. Therefore, it is essential to recognise relations between abundance of questing ticks and factors associated with tick habitat and host pattern. In this study, we investigated these relations for Belgium using a multi-level approach. In general, *I. ricinus* ticks were found widely distributed in the forested areas of Belgium. The abundance of questing nymphs appeared sensitive to more selected variables than adults. The spatial pattern of ticks sampled is similar to that of recorded cases of Lyme disease in Belgium, but not exactly the same [27]. Spatial variations of disease incidence depend not only on the distribution of the vector but also on the land use patterns that may influence human exposure to ticks [28].

Consistent with current knowledge, tick questing patterns in Belgium were influenced by short term weather conditions at sampling level. The dryness of the environment seemed related to different effects on the sampling results at daily and weekly scales.

At the daily level, environment dryness was related to more sampled ticks. However, while relative humidity and vapour pressure deficit were highlighted for nymphs at the sample level, only relative humidity was retained, with a marginal predictive power, in the above-average models. This is in contrast to previous studies [2,3,9]. Similar to our findings, Hubalek et al. [29] and Ruiz-Fons and Gilbert [30] found a negative association between daily abundance of questing nymphs and relative humidity. Tagliapietra et al. [14] also identified an optimal value of 0.98 kPa for vapour pressure deficit below which the effect was positive. While the reason remains unknown for such positive relation between short-term environment dryness and abundance of sampled nymphs, several explanations can be proposed. Firstly, lower vapour pressure deficit may lead to a longer period of questing [4] and a lower mortality rate [31]. This may contribute to a high feeding success of nymphs on their natural hosts and thus reduce the chance of encountering questing nymphs during sampling. Secondly, meteorological data derived from climate stations may lead to uncertainty when used for forested sampling sites more than 10 km away. In a forest ecosystem, the soil and ground-level humidity may be adjusted by ground vegetation or litter [32] and air temperature is modified by canopy [33]. These microclimatic effects can respond to short-term variations of weather conditions. Hence, field measurement of meteorological variables is required to gain more insight into the biology of ticks. Thirdly, humidity gradient within the vegetation layer may also influence the vertical distribution of ticks. The atmosphere is permanently moist near the roots where ticks remain quiescent (mostly shorter than 1 day [4]), but can be generally dryer at the vegetation tips where ticks quest. Ticks will need to rehydrate every now and then to avert desiccation and must return to the moist ground layer periodically to do so. *Ixodes ricinus* nymphs are known to be able to establish “avoiding reactions” [34], i.e. when they go from the tip of vegetation toward the root and approach the vertical humidity boundary, they may move to the dry side to avoid the change of humidity. This may keep ticks near the tip of vegetation till desiccation and thus increase the probability of being sampled.

At the weekly level, drying of the environment seemed to have resulted in a lower abundance of sampled nymphs and adults. Wind speed in the previous 7 days was found to be important in a negative relationship to the daily questing abundance of both nymphs and adults. Similarly, in Texas, a negative relation was found between the average wind speed of the 3 weeks prior to the sampling day and the abundance of immature *Amblyomma cajennense* ticks [35]. Continuous wind may increase the dispersal of carbon dioxide which is an attractant for Ixodid ticks [36]. Other than this, it may fasten the forest air replacement by dryer air from less vegetated areas. Such consequences on a relative long term (i.e. a week) can lead to an increase in tick mortality.

In this study, sampling focused on favourable areas, forests, allowing comparison of specific features of forested landscape structure. Among identified site-level determinants, landscape composition factor (i.e. coverage of broad-leaved forests) seemed to be important to the adult abundance only. However, forest fragmentation was important to the abundance of both nymphs and adults. In general, more ticks were sampled in landscapes where forest is more fragmented, with longer edges and patches that are close to each other. In Belgium, as in many places, these features result from the way landscapes are shaped by human use of the land. In the field, edge density, shape index and fractal dimension are all good indicators to plant species richness [37]. This may be attractive to foraging deer, and may therefore contribute to high local tick abundance. Besides, smaller between-patch distance and lower aggregation index of forest can indicate a higher probability of hosts moving between the forest patches which, in theory, can contribute to a higher tick density in the forest [38,39]. Interestingly, nymphs and adults responded differently to the buffer radii used for the nearest neighbour distance. It appeared to be more important for nymphs at a smaller geographical extent (buffer radius = 500 m, area = 79 ha) and for adults at a larger geographical extent (buffer radius = 1500 m, area = 707 ha). This may be associated to the home ranges of the preferred host for tick at different life stages. The distribution of nymphs can be influenced by the movements of hosts on which larvae feed, whereas the distribution of adults can be influenced by the movements of hosts on which nymphs feed. Larval ticks tend to feed on small-sized animals such as rodents whose home ranges are typically smaller than 79 ha. For example, the home ranges of bank vole and wood mice are around 0.2 ha [40,41]. Nymphs feed not only on rodents but also on larger animals such as roe deer and red deer whose home ranges can be as large as 100 ha and 750 ha respectively [42]. Such differences can advise the design of the environmental data collection schemes for the future empirical works that specifically focus on tick life stages.

Roe deer hunting was not identified as a key determinant to the dynamics of ticks from the multi-level models. The number of roe deer shot was found negatively but marginally associated with questing nymphal abundance in single level regressions only. Despite the fact that what proportion of the total population is shot is unknown, the hunting of roe deer seemed to reduce the abundance of nymphs. Hunting reduces both the roe deer population and the feeding success of adult ticks. This may result in a decrease of local tick population. Besides, hunting activity is performed mostly in relation to forests. Such forests normally contain various and abundant animal species that are suitable to host ticks. As a result, the natural tick feeding success can be high and only a few ticks would be left to be encountered during sampling.

Several potential limitations of the present study should be noted. Firstly, though the abundance of sampled ticks is an important indicator of human exposure to questing ticks, it does not equal the tick density. The site-level sampled results thus cannot adequately reflect the tick density differences in Belgium. Further investigation on the relations between tick abundance to hosts populations and spatial dynamics including movements, would be useful. Secondly, the effects of wildlife management need to be further investigated. Hunting data of 2009 were used as a site-level variable. However, it is likely that there would be inter-annual variations in shooting patterns due to the change of wildlife and land management policy, or failed execution of local shooting plan. Also, the influence of such variation on tick population may appear only after a year or two [43]. Better data on wildlife, including large vertebrates, is therefore crucial for understanding better the links between tick abundance and host abundance. Thirdly, current interpretation of the sampled results can be modified by including microhabitat factors of the sampled area. Even if in the same forest stand, tick abundance has been shown to vary under different topographical, soil and ground vegetation conditions [6,15]. Understanding their effects can in turn contribute to a better standardisation of the sampling procedure in large-scale investigations. Finally, the small group sizes can significantly influence the predictive power of multi-level models [22] and may result in an overestimation of site level variance [44]. Continuing studies for Belgium can be based on the present study and should preferably focus on establishing more seasonal samplings on fewer sites.

## Conclusions

A multi-level approach was performed to study the spatio-temporal dynamics of *I. ricinus* ticks in Belgium. Several environmental determinants were identified for daily abundance of questing ticks including temperature, relative humidity and wind speed at sampling level and forest fragmentation at site level. The study showed that the effects of environmental variables on tick life stages responded differently to different spatial and temporal scales. It can contribute to optimise spatio-temporal data collection schemes for future empirical investigation and to introduce the potential parameters for the future biological tick models.

## Competing interests

The authors declare no competing interests.

## Authors’ contributions

PH, CC and LS carried out the field survey and laboratory work. SL and SV designed the statistical modelling approach. SL performed the statistical analysis of data. SL and SV interpreted the results. SL and SV drafted the first version of the manuscript. All the authors reviewed and approved the final version of the manuscript.
